# Intra-arterial versus intra venous contrast-enhanced computed tomography of the equine head

**DOI:** 10.1186/s12917-016-0632-9

**Published:** 2016-01-07

**Authors:** Casper P. Crijns, Yseult Baeumlin, Lieve De Rycke, Bart J.G. Broeckx, Lieven Vlaminck, Erik H. J. Bergman, Henri van Bree, Ingrid Gielen

**Affiliations:** Department of Veterinary Medical Imaging and Small Animal Orthopaedics, Ghent University, Faculty of Veterinary Medicine, Merelbeke, Belgium; Tierärztliches Überweisungszentrum, Tenniken, Switzerland; Pharmaceutical Sciences, Ghent University, Ghent, Belgium; Surgery and anaesthesiology of large animals, Ghent University, Merelbeke, Belgium; Lingehoeve Diergeneeskunde, Lienden, Netherlands

**Keywords:** Horse, Imaging, Computed tomography, Contrast, Head

## Abstract

**Background:**

The anatomical complexity of the horse’s head limits the abilities of radiography. Computed tomography (CT) in combination with contrast enhanced CT is used more often for diagnosing various head pathology in horses. The objective of this study was to compare intravenous and intra-arterial contrast-enhancement techniques and describe normal and abnormal contrast enhancement in the horse’s head.

**Results:**

All 24 horses included in the study recovered without complication from the procedures. Compared to the pre-contrast studies, post-contrast studies showed significant contrast enhancement in the pituitary gland (IA: *p* < 0.0001; IV: *p* < 0.0001), IA nose septum (*p* = 0.002), nose mucosa (IA: *p* < 0.0001; IV: *p* = 0.02), parotid salivary gland (IA: *p* < 0.0001; IV *p* < 0.0001), cerebrum (IA: *p* < 0.0001; IV: *p* < 0.0001), rectus capitis muscle (IA: *p* < 0.0001; IV *p* = 0.001), IA temporal muscle (*p* < 0.0001), IA masseter muscle (*p* <0.0001) and IV brainstem (*p* = 0.01). No significant contrast enhancement was seen in the eye (IA: *p* = 0.23; IV *p* = 0.33), tongue (IA *p* = 0.2; IV *p* = 0.57), IA brainstem (*p* = 0.88), IV nose septum (*p* = 0.26), IV temporal muscle (*p* = 0.09) and IV masseter muscle (*p* = 0.46). Three different categories of abnormal enhancement were detected: a strong vascularised mass, an enhanced rim surrounding an unenhanced structure and an inflamed anatomical structure with abnormal contrast enhancement.

**Conclusion:**

Using the intra-arterial technique, similar contrast enhancement is achieved using less contrast medium compared to the intravenous technique. And a potential major advantage of the IA technique is the ability to evaluate lesions that are characterized by increased blood flow. Using the intravenous technique, a symmetrical and homogenous enhancement is achieved, however timing is more crucial and the contrast dosage is more of influence in the IV protocol. And a potential major advantage of the IV technique is the ability to evaluate lesions that are characterized by increased vascular permeability. Knowing the different normal contrast enhancement patterns will facilitate the recognition of abnormal contrast enhancements.

## Background

Radiography is a primary ancillary imaging modality for the evaluation of the horse’s head. However, due to anatomical complexity and superimposition of tissues of the head, the interpretation of the radiographs remains difficult [1]. The tomographic ability to produce reconstructed images allows computed tomography (CT) compared to radiography to excel in depicting the complex anatomical structures of the head [2].

Normal CT anatomy of the horse’s head has been described in foals [3] and in adult horses [4]. In horses, CT of the head has been used for the diagnosis of sinusitis, alveolitis and apical infection of the check teeth [5, 6], mandibular, nasal and paranasal tumors and cysts [7–9], ethmoid hematoma [10], parapharyngeal aneurysm [11], fractures [12–14], temporohyoid osteopathy [12], neurologic [15] and intracranial disorders such as abscesses [16], acute haemorrhage and other space-occupying masses [13, 17], and is routinely performed in such cases. Contrast media are used to perform contrast-enhanced CT examinations to accurately locate masses in the head, fully depict the extension of space occupying diseases in these areas and help plan the surgical approach [7, 18, 19]. Contrast enhancement CT after intravascular contrast medium administration is based on the principle of an increased opacity in (neo-) vascularized structures and extra-vascular structures in comparison to the native CT study [20, 21]. In small animals, contrast-enhanced CT of the head is common practice following an intravenous route of contrast administration [22–25]. Contrast-enhanced CT helps in these cases to differentiate normal soft tissues from lesions in the soft tissues as soft tissue lesions show an alteration in their blood flow and/or have an altered vascular permeability [26].

In horses, two routes of intravascular contrast injection have been reported. The most common is the total body systemic intravenous (IV) route as described in small animals. A systemic bolus of contrast medium is administered intravenously; the contrast medium will pass the heart first and will be diluted before reaching the region of interest. In small animals a protocol of 2 mL/kg body weight (~600mgI/kg body weight) of intravenous contrast medium injected at a rate of 2 mL/s has been established [22, 24, 25]. For horses this protocol is more costly and impractical due to the large volume that needs to be injected. In the equine literature several protocols with a wide range of volumes and dosages (250–400 mL of 350–370mgI/mL or about 160–250mgI/kg body weight) have been described for horses [9, 13, 27]. A second route is intra-arterial (IA) contrast enhanced CT, which has been described to characterize distal limb conditions [20, 21] and more recently has been presented for the horse’s head [28]. The advantage of the direct route of intra-arterially administration of contrast medium is a local high vascular concentration in the region of interest. In the publications a standardized protocol using continuous IA infusion at a rate of 2 mL/s while scanning an entire volume (the distal limb or the head) beginning 3 s before initiation of the CT scan, injecting a volume of less than 200 mL is described [20, 21, 28].

The first purpose of the current study is to describe and compare the normal contrast enhancement of the soft tissue structures after systemic IV and direct IA administration of contrast medium. The second purpose is to describe the differences in abnormal contrast enhancement between the two techniques. We hypothesised that 1) after IV or IA contrast administration, post-contrast CT images show significant enhancement of the selected soft tissue structures compared to the pre-contrast CT images, 2) while using less contrast medium with the IA technique, a similar or higher contrast enhancement will be obtained as with the IV technique and 3) both technique would allow to detect some specific abnormal contrast enhancement patterns.

## Methods

### Study design

Retrospective comparative clinical study, ethical committee oversight is currently not required for this type of study.

### Cases

Case records were reviewed for horses, which underwent native CT and intravascular (IV or IA) contrast-enhanced CT evaluation of the head. All horses that underwent IA contrast-enhanced CT were included. A selection of IV contrast-enhanced cases was made based on the present of a complete CT evaluation of the head.

### Computed tomographic examination

CT scans of the head were acquired with the horses under general anaesthesia. Each horse was sedated using detomidine^1^ (0,005 mg/kg) as a premedication. Anaesthesia was induced with a combination of ketamine^2^ (2,2 mg/kg, IV) and midazolam^3^ (0,06 mg/kg, IV). After intubation, anaesthesia was maintained with inhaled isoflurane^4^ and oxygen (on effect, ±1,2 % expiratory). Vital parameters like heart rate, ECG, respiratory rate and respiratory curve were monitored continuously.

The horses were positioned in dorsal recumbency with the head in the gantry. Images were acquired with two fourth generation 4-slices helical CT scanners (Lingehoeve Equine Clinic: Philips Mx8000 Quad multislice CT scanner^5^; Ghent University: GE Medical Systems Prospeed four slice^6^) using the in-house protocols for equine head imaging (Lingehoeve Equine Clinic: 120 kV, 185mAs, pitch 1, 1,3 mm slices, 512x512 matrix, scan FOV 293–463 mm, sharp algorithm; Ghent University: 120 kV, 160mAs, pitch 1, 1,25–2,5 mm slices, 512x512 matrix, scan FOV between 192 and 403 mm, bone and standard algorithm).

A pilot study was performed first to assess the symmetric position of the horse’s head in the gantry, followed by pre- and post-contrast CT studies in all horses.

### IA contrast protocol

IA contrast-enhanced CT studies were performed after aseptic ultrasound guided (7,5mHz linear probe) catheterization (14 gauge x 80 mm) of one of the common carotid arteries in the mid to caudal cervical region through the skin, with the bevel directed towards the heart. The common carotid artery was located deep to the jugular vein and color Doppler helped to confirm the arterial nature of the blood vessel. The catheter was introduced trough the thick wall of the carotid artery in one fast and strong movement. The catheter was fixed to the skin with polypropylene sutures. A three-way stopcock and an extension set were attached to the catheter to facilitate injection. The extension set was prefilled with contrast medium before attaching to the catheter. A pressure injector containing 250 ml of non-ionic iodinated contrast medium was connected to the extension set. A continuous arterial infusion protocol during scanning was used, with an injection rate of 2 mL/s (total volume injected <180 mL) of Ioversol 350mgI/mL (Optiray®350^7^). The post-contrast scan was initiated with a 3 s delay from the start of the contrast medium administration. The intra-arterial catheter was removed at the end of the procedure and a 5-min manual compression was applied to avoid bleeding and hematoma formation. Then a bandage with moderate compression was placed around the neck at the site of injection for the time of the recovery.

### IV contrast protocol

IV contrast-enhanced CT studies were performed after blind catheterization (18 gauge x 80 mm) of one of the cephalic veins in the thoracic limbs, with the bevel directed away from the heart. The catheter was fixed to the skin with superglue. Two pressure injectors containing 200 ml of non-ionic iodinated contrast medium were connected to an extension set. The extension set was prefilled with contrast medium before attaching to the catheter. A systemic bolus of 400 mL of Iobitridol 350mgI/mL (Xenetix®350^8^) was injected at a rate of 15 mL/s. The post-contrast scan was initiated about 30s after the start of the contrast medium administration. After removal of the catheter a bandage with moderate compression was placed around the limb at the site of injection for the time of the recovery.

### Image analysis

**Fig. 1:**
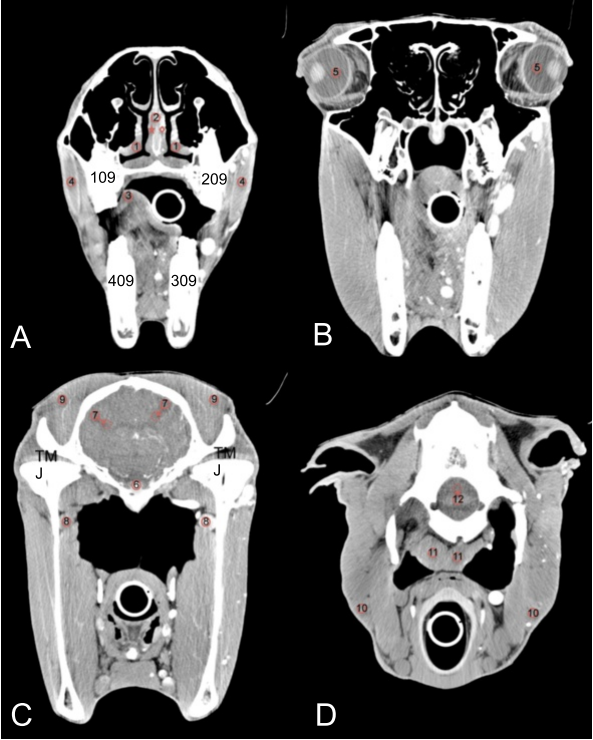
Transverse IA contrast enhanced CT images of the equine head (right is to the left and dorsal is to the top). **a** At the level of the 09’s, ROI’s are placed on the epithelial lining of the ventral nasal conchae (1), the nasal septum (2), the tongue (3) and the masseter muscles (4). Note the homogeneous (star) and inhomogeneous and patchy (diamond) enhancement pattern in the nasal septum. **b** At the level of the eyes, ROI’s are placed on the corpi vitreum (5). **c** At the level of the temporo-mandibular joints, ROI’s are placed on the pituitary gland (6), the cerebral cortex (7), the maxillary veins (8) and the temporal muscles (9). **d** At the level of foramen magnum, ROI’s are placed on the parotid salivary gland (10), the longus capitis muscles (11) and the brainstem (12). Note the different ROI’s placed on the grey matter (circle) and white matter (doted circle) of the cerebrum and brainstem in figures (**c**) and (**d**)

**Fig. 2:**
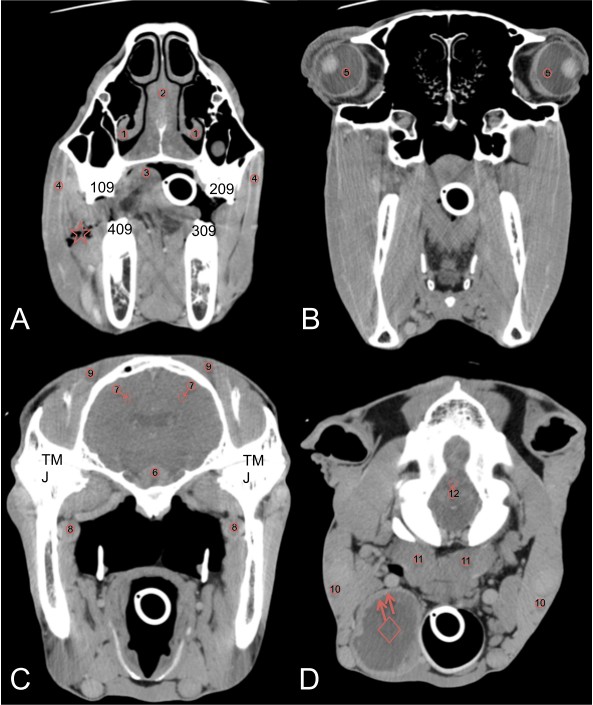
Transverse IV contrast enhanced CT images of the equine head (right is to the left and dorsal is to the top). **a** At the level of the 09’s, ROI’s are placed on the epithelial lining of the ventral nasal conchae (1), the nasal septum (2), the tongue (3) and the masseter muscles (4). Note the air opacities (star) in the mild enhancing and swollen right masseter muscles. **b** At the level of the eyes, ROI’s are placed on the corpi vitreum (5). **c** At the level of the temporo-mandibular joints, ROI’s are placed on the pituitary gland (6), the cerebral cortex (7), the maxillary veins (8) and the temporal muscles (9). **d** At the level of foramen magnum, ROI’s are placed on the parotid salivary gland (10), the longus capitis muscles (11) and the brainstem (12). Note the rim enhancement (arrows) surrounding a hypodense retropharyngeal abcess (diamond). Note the different ROI’s placed on the grey matter (circle) and white matter (doted circle) of the cerebrum and brainstem in figures (**c**) and (**d**)

Image analysis was performed separately by two of the authors on a dedicated diagnostic imaging viewing station with a 2880x1800 pixel flat screen monitor using Osirix^9^ DICOM viewer software. Images were reviewed using a soft tissue (window wide (WW): 200–400 Hounsfield Unit (HU); window level (WL): 40–100 HU) and a bone window (WW: 2000 HU; WL: 750 HU). The post-contrast images were evaluated using a soft tissue window to review the normal pattern of contrast enhancement and to describe the abnormal enhancement due to pathology. A semi-quantitative grading scale was used to describe (abnormal-) contrast enhancement in the different soft tissue structures: none (no enhancement), mild (minimal enhancement), moderate (apparent enhancement) and severe (obvious enhancement). Circular semi-automatic region-of-interest (ROI’s) (size: ~0,5 cm^2^) were manually placed on predefined normal soft tissue structures on the contrast enhanced images, as illustrated in Figs. 1 and 2 using the sharp (IA studies) and standard (IV studies) algorithm. To increase to repeatability of the ROI placement, only easily identifiable soft tissue structures were reviewed in this study, on which the ROI’s could be placed completely isolated from adjacent structures. To ensure that the ROIs contained mainly the parenchyma of the soft tissue structures, we avoided inclusion of macroscopic vessels visible in the structures. The areas of interest in the soft tissue structures were selected on 4 different transverse slides. The most rostral selected slide at the level of the first molars (Figs. 1a and 2a), allowed reviewing the epithelial lining of the nasal conchae, the nasal septum and the tongue as well as the body of the masseter muscle. On the second slide, at the mid level of the orbita the corpus vitreum of the eyes was reviewed (Figs. 1b and 2b). The third slide was selected at the level of the temporomandibular joint (Figs. 1c and 2c), as at this level the pituitary gland is anatomically situated, allowing the review of the pituitary gland, the cerebral cortex, the maxillary vein and the body of the temporal muscle. The most caudal slide was selected at the level of foramen magnum: the brainstem, the parotis salivary gland and the body of the longus capitis muscle are easily identified at this level (Figs. 1d and 2d). To assure the ROI’s on the pre- and post contrast images were incorporating the same tissue, the ROI’s were copied and pasted to the pre-contrast images. The mean value registered in the ROI’s was recorded and used for the quantification of contrast enhancement in the selected normal soft structures. If structures were not incorporated in the CT study due to a partial study of the head or if a structure was obliterated by pathology e.g., due to a mass effect, ROI’s were not placed on these structures.

### Statistical analysis

A mixed model analysis was conducted for each separate anatomical structure (dependent variable).^10^ Independent fixed effects were the effect of observer (one or two), contrast medium (yes or no), route of contrast medium administration (IV or IA) and, if the structure was measured bilaterally, asymmetry (side of the catheter or contralateral side). Patient was included as random effect. Homoscedasticity and normality of residuals were visually checked using residuals versus fitted plots and QQ plots. If necessary, power transformations were applied, using a Box-Cox plot for guidance. A *P*-value <0,05 (linear mixed model) was considered significant.

## Results

Eleven warmblood horses and 1 Arabian horse that underwent IA contrast-enhanced CT of the head at Lingehoeve Equine Clinic were identified, 7 geldings, 3 mares and 2 stallions, median age of 8,5 years (range between 1 and 18 years). A group of 12 horses (10 warmblood horses, 1 trotter and 1 pony) that underwent IV contrast-enhanced CT evaluation of the complete head at the Department of Medical Imaging of the Faculty of Veterinary Medicine of Ghent University were selected for the study. Four geldings, 7 mares and 1 stallion, median age 14 years (range between 4 and 25 years), median weight 540 kg (range between 375 and 695 kg).

The CT diagnoses of the included cases were: apical infections in combination with secondary sinusitis (*n* = 6), ethmoid hematoma (*n* = 4), sinus cyst (*n* = 3), mass/neoplasm (*n* = 2), inflammatory/infectious disease (*n* = 2), traumata (*n* = 1) bilateral temporohyoid osteoarthropathy (*n* = 1) and no diagnosis (*n* = 5) (Table 1).

**Table 1:** Anamnesis of the horses included in the study, contrast administration protocol used and final (CT) diagnosis

Case	Breed	Age	Sex	Reason to perform the scan	IV/IA	Diagnosis	Enhancement
1	WB	17	G	Intermittent unilateral epistaxis	IA 2 mL/s	No significant abnormalities	x
2	WB	8	M	Headshaking	IA 2 mL/s	Widened infraorbital canal, remaining tooth fragments	x
3	WB	4	G	Unilateral nasal discharge	IA 2 mL/s	Ectopic tooth, apical infection	x
4	WB	1	M	Facial swelling, difficulty breathing	IA 2 mL/s	Sinus cyst	Mild enhancement of the capsule
5	WB	4	G	Facial swelling, unilateral nasal discharge	IA 2 mL/s	Inflammatory/infectious disease with intra-cranial extension	Irregular enhancement of the trigeminal nerve and soft tissue enhancements surrounding the osteolysis
6	Arabian	11	S	Soft tissue mass soft palate	IA 2 mL/s	Squamous cell carcinoma	Heterogeneous enhancement of the mass
7	WB	14	G	Swelling behind left mandible	IA 2 mL/s	Soft tissue mineralisation’s, bilateral TMJ disease,	x
8	WB	18	S	Bilateral sinusitis	IA 2 mL/s	Progressive ethmoidal hematoma	Very mild enhancement
9	WB	8	M	Infectious process	IA 2 mL/s	Fractured right external acoustic meatus	x
10	WB	9	G	Opacity in the frontal sinus	IA 2 mL/s	Sinus cyst	x
11	WB	6	G	Unilateral nasal discharge	IA 2 mL/s	Apical infection, tooth fracture and secondary sinusitis	x
12	WB	1	G	Facial swelling, unilateral nasal discharge	IA 2 mL/s	ameloblastoma	x
13	WB	10	M	Epistaxis after surgery	IV 241 mgI/kg	Subcutaneous emphysema	x
14	WB	19	M	Study	IV 305 mgI/kg	No significant abnormalities	x
15	Trotter	22	G	Unilateral nasal discharge with decreased air passage	IV 281 mgI/kg	Progressive ethmoidal hematoma with secondary sinusitis	Adjacent mucosal enhancement
16	WB	8	M	Unilateral nasal discharge	IV 216 mgI/kg	Apical infection with abscess	Mild enhancement of the capsule
17	WB	16	M	Intermitted unilateral epistaxis	IV 311 mgI/kg	Progressive ethmoidal hematoma	Very mild enhancement
18	WB	9	G	Difficulty breathing	IV 252 mgI/kg	Sinus cyst	Mild enhancement of the capsule
19	Pony	4	S	Facial swelling	IV 373 mgI/kg	Alveolitis	x
20	WB	25	G	Intermitted unilateral epistaxis	IV 201 mgI/kg	Progressive ethmoidal hematoma	Rim enhancement
21	WB	10	M	Unilateral nasal discharge	IV 219 mgI/kg	Alveolitis with secondary sinusitis	x
22	WB	16	M	Epilepsy	IV 257 mgI/kg	No significant abnormalities	x
23	WB	9	M	Epilepsy	IV 260 mgI/kg	No significant abnormalities	x
24	WB	22	G	Maxillary swelling	IV 279 mgI/kg	Inflammation of the right masseter musc. With abcess	Mild homogeneous enhancement of the musc and rim enhancement surrounding an abcess

The first attempt catheterization of the common carotid artery was not fully successful leading to hematoma formation. The reason of the hematoma formation was a hesitant introduction of the catheter through the thick arterial wall and probably dissection of the wall with leakage of blood from the arterial lumen. The procedure was still pursued and no harmful side effects were observed for the patient.

All horses recovered without complications, no adverse reactions on the contrast medium administrated or administration technique were observed in the included patients.

### Normal contrast enhancement

#### IA protocol

**Fig. 3:**
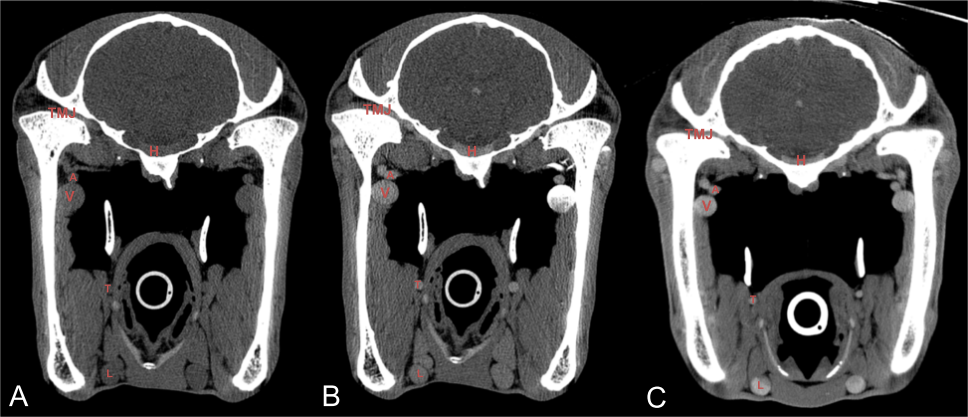
Transverse pre-contrast (**a**), IA post-contrast (**b**) and IV post-contrast (**c**) CT images at the level of the temporo-mandibular joints (right is to the left and dorsal is to the top). **a** The visibility of the vascular structures are less clear definite compared to the post-contrast images, especially the smaller arterial structures (A and T) are more difficult to distinguish from the surround soft tissue. **b** Bilateral contrast-enhancement after unilateral left-sided intra-arterial contrast injection is clearly delineating the vascular structures; contrast streaming is seen in the left maxillary vein. **c** Homogenous, bilateral contrast-enhancement of the arterial and venous vascular structures after intra-venous contrast injection. TMJ: temporomandibular joint; H: pituitary gland; A: maxillary artery; V: maxillary vein; T: truncus linguofacialis; L: linguofacial vein

During the IA injection of contrast medium, there was moderate to severe contrast enhancement of the maxillary veins (Table 2). In this group, the contrast enhancement was asymmetrical between injection side and the contralateral side in all cases (Figs. 1 and 3). In the maxillary veins ipsilateral to the side of intra-arterial injection, part of the contrast medium was seen pooling along the dependant wall of the vein creating a layered and partial severe contrast enhancement in the lumen of the vein in 7 of the 12 cases. In 4 of these patients this layer of non-mixed contrast medium occupied about 50 % of the lumen. In the other 3 patients the layer of contrast medium occupied about 25 % of the lumen of the vein. In the maxillary vein contralateral to the side of injection, no such dependant contrast medium layer was detected in any case. Enhancement in this vein was considered moderate and homogeneous (Table 2, Fig. 3).

**Table 2:** The median and range of the HU measurements of the different structures, ipsilateral and contralateral of the injection site, on the pre- and post-contrast CT images for the intravenous and intra-arterial protocols

Structure	Injection Side	Intravenous Protocol			Intra-arterial Protocol		
		n	Pre-Contrast (HU)	Post-Contrast (HU)	n	Pre-Contrast (HU)	Post-Contrast (HU)
Maxillary vein	Ipsilat.	12	63.8(34.9–90.7)	109.7(81.3–146.5)	12	62.3(43.2–97.1)	307.9(91.8–2587.6)
	Contralat.	12	63.0(36.7–97.8)	104.3(67.9–151.4)	12	61.0(48.7–80.7)	112.6(66.6–241.4)
Pituitary gland	N/A	12	47.5(38.5–66.6)	78.7(58.6–100.0)^a^	12	57.2(34.7–108.0)	83.8(56.5–182.2)^a^
Nose septum	N/A	9	68.5(32.5–115.0)	81.9(61.9–120.9)	11	67.6(50.5–108.1)	97.3(63.2–142.4)^a^
Nose mucosa	Ipsilat.	9	64.0(24.8–134.5)	83.7(23,3–142.5)^a^	10	48.2(40.0–92.4)	77.7(40.9–125.5)^a^
	Contralat.	8	63.2(14.4–128.6)	89.8(36.3–127.3)^a^	10	56.6(27.3–98.6)	86.1(30.3–148.0)^a^
Parotis salivary gland	Ipsilat.	12	44.7(24.3–61.1)	62.0(37.6–97.7)^a^	8	42.0(32.4–55.5)	62.9(37.2–95.6)^a^
	Contralat.	12	47.7(32.9–61.7)	66.3(37.8–110.5)^a^	8	43.7(33.7–53.8)	65.5(32.3–93.7)^a^
Cerebrum	Ipsilat.	12	40.0(30.5–55.0)	41.4(29.8–56.4)^a^	12	38.2(31.4–53.8)	45.7(33.2–64.4)^a^
	Contralat.	12	40.5(30.2–53.0)	44.8(31.4–56.1)^a^	12	38.7(30.4–67.0)	45.8(35.2–91.1)^a^
Longus capitis musc.	Ipsilat.	12	46.3(19.6–59.8)	52.1(23.6–65.1)^a^	8	52.2(30.7–58.9)	57.3(43.5–95.3)^a^
	Contralat.	12	50.8(32.9–66.4)	54.6(36.0–67.1)^a^	8	51.1(35.7–57.8)	62.0(50.3–83.9)^a^
Temporal musc.	Ipsilat.	12	66.5(47.7–87.7)	69.3(50.0–91.8)	12	68.4(46.9–81.7)	73.7(59.4–91.0)^a^
	Contralat.	12	66.1(44.6–79.6)	67.8(49.8–97.0)	12	67.3(47.3–76.6)	75.3(55.2–92.0)^a^
Masseter musc.	Ipsilat.	9	79.4(50.8–125.8)	85.3(53.5–140.0)	11	85.5(55.6–98.6)	91.0(67.8–154.5)^a^
	Contralat.	9	79.3(55.5–128.6)	83.0(59.8–129.0)	11	84.7(52.4–105.4)	84.9(59.7–149.6)^a^
Corpus vitreum	Ipsilateral	11	14.9(4.2–25.9)	15.7(8.3–25.8)	11	9.7(2.7–20.9)	11.2(3.3–24.8)
	Contralat.	12	15.3(4.6–28.8)	14.8(5.6–23.4)	11	8.7(2.8–21.0)	8.5(1.5–39.8)
Brainstem	N/A	12	44.7(31.6–64.4)	48.3(33.6–67.4)^a^	8	39.8(23.0–68.0)	40.0(21.5–75.2)
Tongue	N/A	9	55.7(23.2–103.2)	62.2(24.4–107.4)	11	48.4(17.8–78.4)	56.3(27.7–81.2)

*n* amount of structures measured, *HU* Houndsfield Unit; ^a^ Significant increase in contrast enhancement between pre- and post contrast scans

Mild to moderate contrast enhancement was seen and measured for the pituitary gland (*p* < 0.0001), nose septum (*p* = 0.002), nose mucosa (*p* < 0.0001) and parotid salivary gland (*p* < 0.0001) (Table 2). Two different enhancement patterns of the nose septum and nose mucosa were observed in the included cases. In 7 cases the enhancement was mild, homogeneous and symmetrical. In 2 cases the enhancement was inhomogeneous, patchy and not always symmetrical (Fig. 1).

On the post-contrast scans the cerebral arteries became asymmetrical and more clearly visible on the ipsilateral side of injection during IA contrast administration (Fig. 1). The cerebrum showed only none to mild contrast enhancement (*p* < 0.0001). None to mild contrast enhancement was also only measured, but not detected while visually reviewing the cases for the rectus capitis muscles (*p* < 0.0001), temporal muscles (*p* < 0.0001) and masseter muscles (*p* = 0.001).

No significant contrast enhancement was detected in the corpus vitreum (*p* = 0.23), the brainstem (*p* = 0.88) and the tongue (*p* = 0.12) (Table 2).

#### IV protocol

After IV injection of contrast medium, moderate, homogeneous and symmetric between the right and left side in all cases, independently of the catheter side was detected (Figs. 2 and 3) and measured (Table 2).

Mild to moderate contrast enhancement was seen and measured for the pituitary gland (*p* < 0.0001), nose mucosa (*p* = 0.02) and parotid salivary gland (*p* < 0.0001) (Table 2). Mild to moderate contrast enhancement was only seen and measured in the nose septum, but this enhancement was not significant (*p* = 0,26) in the entire group. Two different enhancement patterns of the nose septum and nose mucosa were also observed in this group. In 7 cases the nose mucosa and in 5 cases the nose septum showed mild, homogeneous and symmetrical enhancement (Fig. 2). In 4 cases the enhancement was inhomogeneous, patchy and not always symmetrical. And in 2 cases the nose septum showed no clear enhancement.

On the post-contrast scans the cerebral arteries became mild and symmetrical visible after IV injection of contrast medium (Fig. 2). The cerebrum was only none to mild contrast enhancing (*p* < 0.0001) (Table 2). None to mild contrast enhancement was also only measured, but not detected while visually reviewing the cases for the rectus capitis muscles (*p* = 0.001) and the brainstem (*p* = 0.01) (Table 2).

No significant contrast enhancement was detected in the corpus vitreum (*p* = 0.33), temporal muscles (*p* = 0.09), masseter muscles (*p* = 0.46) and the tongue (*p* = 0.57) (Table 2).

#### Comparing the IA and IV protocol

The eye (*p* = 0.02), the pituitary gland (*p* = 0.05), longus capitis muscles (*p* = 0.02) showed a significant higher increase in attenuation for the IA protocol compared to the IV protocol (Table 2).

#### Comparing the two observers

A significant difference in measurements between the two observers was detected for the cerebrum (*p* < 0.0001) and brainstem (*p* < 0.0001) only (Figs. 1 and 2).

### Abnormal contrast enhancements

**Fig. 4:**
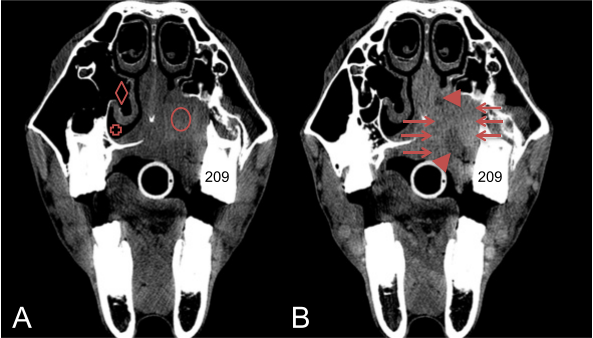
Transverse CT and IA contrast enhanced CT images of case 6 (right is to the left and dorsal is to the top). **a** Squamous cell carcinoma in the hard palate causing a mass effect in the left ventral nasal meatus (cross) and suppressing the left ventral conchal sinus (diamond). The hard palate and the alveolar bone of elements 209 and 210 are destructed. **b** Heterogeneous contrast enhancement of the mass, enhancement of the mass is indicated by the arrows and non-enhancing portion of the mass are indicated by arrowhead

**Fig. 5:**
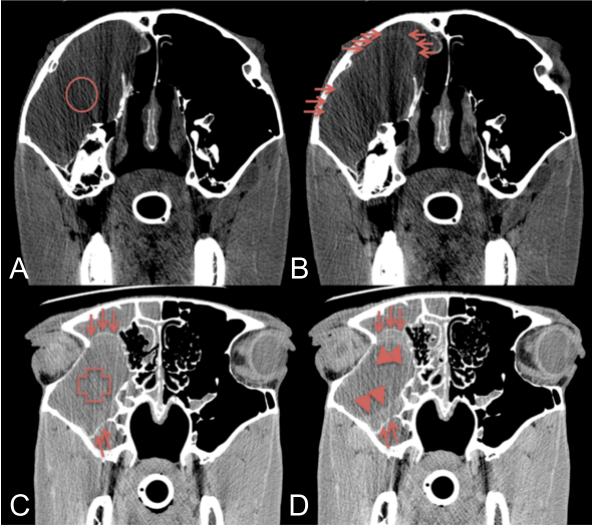
Transverse CT and IV contrast enhanced CT images of two cases with a sinus cyst (right is to the left and dorsal is to the top). **a** Native CT image showing a fluid filled right caudal maxillary and conchofrontal sinus (circle). **b** Contrast enhanced CT image at the same level as image A, showing a thin mild contrast enhancement of the capsule (arrows). **c** Native CT image showing a fluid filled right caudal maxillary and frontal sinus (cross), a partial mineralised capsule is detected (arrows). **d** Contrast enhanced CT image at the same level as image C, showing the partial mineralised capsule (arrows) with an adjacent moderate contrast enhancement (arrowheads) revealing a much thicker capsule

**Fig. 6:**
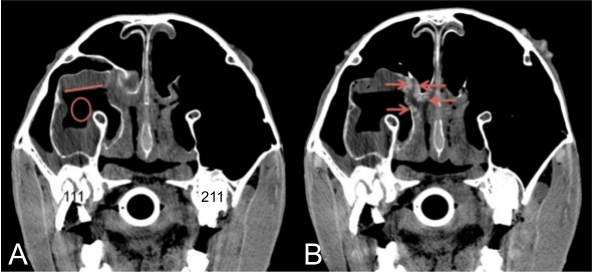
Transverse CT and IV contrast enhanced CT images of case 15 (right is to the left and dorsal is to the top). **a** Secondary sinusitis in the right ventral conchal sinus (circle) characterised by mucosal thickening and free fluid (fluidline). **b** Adjacent mucosal contrast enhancement (arrow heads) is detected

Abnormal contrast enhancement was reported in 4 cases after IA contrast medium administration and in 6 cases after IV contrast medium administration (Table 1). In these reported cases, the detected abnormal contrast enhancements could be divided in three categories: a strong vascularised mass (Fig. 4), an enhanced rim surrounding an unenhanced structure (Figs. 2d and 5) and a normal but inflamed anatomical structure highlighting a region with increased vascular perfusion and permeability (Fig. 6). No differences in these abnormal contrast enhancement patterns were identified between the IV and IA protocols.

## Discussion

In this study the use of IA and IV contrast enhanced CT of the equine head was described using clinical cases. Several studies have described the use of contrast medium in horses [9, 13, 20–22, 26, 27], however the different routes of contrast administration have not been compared. As we used two different scanners in this study, interscanner variability needed to be considered [29].

The absolute attenuation values of the studied structures were only used to determine the structures enhancement and are not directly compared between the two scanners. The contrast medium administration protocols and scanners were consistent pairs. In the presence of interscanner or intrascanner variability, significant differences in enhancement can therefore not be explained without considering differences between the two protocols.

It was hypothesized that, the IA contrast medium administration technique would result in a similar or higher contrast enhancement using a lower volume of contrast medium compared to the IV technique. Contrast enhancement of a structure is intrinsically depending on its specific anatomy and vascular supply, extrinsically on the local dose of contrast medium and the timing of injection relative to the scan protocol. In the described study no controls were performed to exclude the influence of the individual case’s intrinsic properties on contrast enhancement of the different structures. Recognising this shortcoming, we assume differences in contrast enhancement was only influenced by the extrinsic properties.

The local contrast medium dose compared to the total dose administered and the influence of timing are different for the two techniques. The local contrast medium dose in the IV technique is influenced by the amount of contrast medium injected as a bolus and the systemic dilution (influenced mainly by the size of the individual in combination with blood pressure) of this bolus. In the IA technique the dose of contrast medium in the common carotid artery is only influenced by the local dilution with the blood in the common carotid artery at the moment of injection.

Timing of injection relative to the scan protocol for the IV technique is crucial as selective arterial enhancement, the so called “first pass”, is only seen for a relative short period. In human a 10 s time-window of selective arterial enhancement of the carotid arteries was identified 20 s after the start of the intravenous contrast medium bolus administration [30]. The results of the present study showed semi-quantitative moderate to severe enhancement of both the arterial and the venous system in all IV cases. The continuous injection of the IA protocol is assuring the visibility of contrast medium in the arterial lumen. As previously seen for the distal limb, the short delay before initiation of the scan and the time needed to scan the entire head is assuring contrast enhancement of the vascular bed, possible extravascular structures [20, 21] and as shown in the results the venous system. Based on these findings, both techniques represent a mixed (arterial and delayed)-phase CT angiography.

Our results showed for both techniques a similar semi quantitative ranking from highest to least enhancing structures. Highest enhancement and extreme outliers were seen in the maxillary veins. Due to these extreme outliers, statistical analysis was not considered useful. Streaming was the cause of these outliers in the maxillary veins. Streaming is visible as the layered appearance of highly attenuating contrast medium and blood in the vasculature due to the lack of optimal mixing. This phenomenon was detected in both the arterial and venous systems in the IA group. In the arterial system, streaming has previously been reported after intra-arterial drug administration and depends on the rate of infusion, the type of catheter used and the position of the catheter in relation to the arterial branching [31]. Streaming in the venous system has previously been reported to occur in the portal vein following direct injection of contrast medium in the splenic parenchyma in dogs [32]. However, in several cases streaming was present in the venous system and absent in the arterial system after intra-arterial contrast administration. An explanation is lacking for this observation.

The major arteries that supply blood to the head are the both common carotid arteries (left and right side of the head) and the basilar artery (brainstem, cerebellum and caudal cerebrum) [33–35]. Both protocols differ based on the arterial supply of contrast rich blood to the head. The intravenous protocol is non-selective, using both common carotid arteries and the basilar artery, compared to selective intra-arterial protocol, using only one common carotid artery. As only in the IV group a small statistical significant difference in enhancement of the brainstem was measured in this study, the ability of the IA protocol to supply contrast rich blood to the brainstem, cerebellum and caudal cerebrum should be considered a potential limitation of the protocol. However in horses, in contrast to other domestic animals and humans, the common carotid arteries are divided into three arteries (external carotid and internal carotid but also the occipital artery). Via the occipital artery, that is forming the cerebrospinal artery, contrast medium injected in the common carotid artery is supplying the basilar artery in horses [33–35]. Further research will be needed to determine to amount of contrast medium reaching the basilar artery through this connection.

Interestingly, homogenous contrast enhancement of the nose mucosa, parotis salivary gland, cerebrum, temporal muscles and masseter muscles was seen and/or measured in the IA group at the contralateral side following unilateral contrast medium injection. This simultaneous opacification is most likely the result of communications between the right and left arterial systems, through the caudal intercarotid artery and via the arterial circle of Willis [33–36]. Regardless of the side of injection, the nasal mucosa showed a significant enhancement bilaterally. As such, horses with bilateral sinonasal disorders could possibly be scanned following a single arterial injection.

The pituitary gland, the nose septum and mucosa and the parotid salivary gland are highly vascularised structures, which showed marked enhancement on most post-contrast images. Previous reports described the accuracy of delineating the pituitary gland of adult horses, using 250 mL MD-76 (370mgI/mL) [27]. The higher dose of IV contrast medium used in this study, 400 mL Iobitridol (350mgI/mL), will probably not improve one’s ability to detect the pituitary gland and the other marked enhanced structures. However, for the nose septum and mucosa, two patterns of contrast enhancement were observed. In our experience the patchy pattern is more often visible with an increasing dose of contrast medium. Due to a local higher contrast medium concentration, this pattern of enhancement is most likely highlighting the small calibre blood vessels running in these structures. Interestingly, no significant enhancement of the nose septum in the IV group was detected, review of the data showed almost no enhancement in several cases and moderate enhancement in the other cases. Similar results were seen for the temporal and masseter muscles. The absence of significant enhancement in these structures is most likely caused by the variation in contrast dosage (due to the differences in bodyweight) between the individuals included in this group.

The remaining structures had none to very mild significant contrast enhancement. In none of these structures, the enhancement was appreciated while reviewing the cases. Increasing the local dose of contrast medium would theoretically result in higher enhancements in these structures. Based on our results the absence of visible contrast enhancement in these structures after contrast medium administration using one of the above-described protocols has to be considered normal.

A conspicuous finding in these un-enhancing structures is several higher attenuation measurements on pre-contrast images compared to post-contrast images. This is most likely due to small movements of the horses in between scans. The copied ROI’s incorporated therefore not exactly the same tissue samples of the structures. This intra-patient variability has to be considered if abnormal contrast enhancement is diagnosed.

Comparing both techniques, the results display different enhancements in three structures: the eye, pituitary gland and rectus capitis muscle. Although all three structures show a significant difference, for the eye and pituitary gland, the median difference was very small (1.1 and 0.4 HU respectively). For the rectus capitis muscle the difference was higher (6.9 HU). This muscle receives its blood supply directly from the common carotid arteries. A possible explanation for the detected difference could be the streaming causing a local exceptionally high concentration of the contrast medium in the blood supplied to the rectus capitis muscles.

Significant interobserver differences were only detected for the measurements performed on the brain and brainstem. Placement of the ROI’s on these structures differed between the two observers. Reviewing the ROI’s placed on the brain did not allow to detect a difference in attenuation (median interobserver difference of 4.5 HU), this in accordance with previous reports [4]. In contrary depending on the WW and WL setting used to review the brainstem, the grey and white matter could clearly be distinguished (median interobserver difference of 9.2 HU). Depending on the distance to the cranium for the brain and the central or peripheral localisation for the brainstem, an attenuation measurement is made of the more attenuating grey matter (observer 2) or less attenuating white matter (observer 1).

In this study, mild or marked abnormal contrast enhancement was seen in several cases. Three different abnormal enhancement patterns where detected in the included cases: enhancement due to the strong vascularisation of a soft tissue mass, rim enhancement of an encapsulated structure and increased enhancement due to inflammation of an anatomical structure. Diagnostically these findings help in the characterisation of a lesion and in the delineation of the margins between normal soft tissue and soft tissue lesions that are not always evident on pre-contrast images [7, 13, 19]. Especially contrast enhancement in structures, that are normally not or only very mild enhancing, as previously described for cerebral lesions [13, 15] and seen in case 5 and 24, have to be considered abnormal. In none of the cases with abnormal contrast enhancement both techniques were simultaneously performed, therefor-direct comparison between the two protocols in demonstrating specific lesions is not possible based on this study.

Some concerns may be raised for both techniques. In the first case were catheterization of the common carotid artery was attempted this leaded to hematoma formation. The wall of the common carotid artery is thick and the introduction of the catheter should therefor be done with a fast and strong movement to penetrate the vessel wall. This procedure had a steep learning curve considering the fact that only the first catheterization was followed by the formation of a hematoma. A specific concern for intra-carotid drug administration in humans is cerebral embolism due to air emboli [31]. Carefully removing air from the pressure injector and prefilling the extention set prior to attaching to the catheter are provisions to prevent air emboli. In human, a transient but clinically tolerable increase in intra-arterial pressure of the internal carotid and vertebral artery has also been observed, following prolonged intra-arterial injection of contrast medium [37]. In horses, an elevated mean arterial blood pressure or heart rate were seen in 5 % of the cases after intra-arterial iodinated contrast medium administration without requiring intervention [38]. Intra-arterial pressure was not monitored quantitatively in any of the horses included in the study. Although volatile agent-induced hypotension is well known and a concern during inhalation anaesthesia in horses [39]. Our main consideration not to monitor intra-arterial pressure was to keep the total anaesthesia time as short as possible.

A second concern is the contrast medium induced anaphylactic reactions, reported for several species including horses [38, 40–42]. Mild reactions as an elevated heart rate, changes in blood pressure, urticarial and oedema are the most often seen symptoms. Excluding these reactions in the study population solely based on the clinical records is difficult, as for these conditions no treatment or intervention is considered necessary [38, 42] and blood pressure is not standardly recorded in our institutes during CT studies. Moderate and severe anaphylactic reactions require treatment or intervention [38, 42]. No such reactions have been described in the anaesthesia and clinical records of the included cases.

## Conclusions

Either protocol used in this study showed similar marked, mild or none obvious contrast enhancement depending on the reviewed structure. The major advantage of IA contrast medium administration during CT studies is that a similar contrast enhancement is achieved with less contrast medium compared to IV contrast administration, with the disadvantage of the presences of contrast streaming. And a potential major advantage of the IA technique is the ability to evaluate lesions that are characterized by increased blood flow. The major advantage of the IV contrast medium administration in the cephalic vein is the symmetrical and homogenous enhancement, however timing is more crucial and the contrast dosage is more of influence in this protocol. And a potential major advantage of the IV technique is the ability to evaluate lesions that are characterized by increased vascular permeability. Knowing the different normal contrast enhancement patterns of the soft tissues will facilitate the recognition of abnormal contrast enhancements in the horse’s head. Further research will be needed to identify indications specifically profiting from either technique.

## Abbreviations

CT: Computed tomography
IV: Intravenous
IA: Intra-arterial
WW: Window wide
HU: Hounsfield Unit
WL: Window level
ROI: Region of interest

## Footnotes

1 Domidine, Eurovet, Belgium

2 Anesketin, Eurovet, Belgium

3 Dormicum, Roche, Belgium

4 Isoflo, Abbolt Laboratories Ltd, UK

5 Philips Medical Systems, Eindhoven, The Netherlands

6 GE Medical Systems, Milwaukee, Wisconsin, USA

7 Optiray 350, Covidien AG, Hazelwood, Missouri, USA

8 Xenetix 350, Guerbet, Roissy, France

9 OsiriX, Open Source, http://www.osirix-viewer.com

10 Bates D., Maechler M., Bolker B. And Walker S. (2014). Ime4: linear mixed-effects models using Eigen and S4. R package version 1.1.6. http://CRAN.R-project.org/package=lme4

## References

[1] Tremaine W, Dixon P (2001). A long-term study Of 277 cases of equine sinonasal disease. part 1: details of horses, historical, clinical and ancillary diagnostic findings. Equine Vet J.

[2] Rl T, Farrell E (2001). Computed tomography and magnetic resonance imaging of the equine head. Vet Clin N Am-Equine.

[3] Je S, Bc W, Taylor E, Tate L (2002). Anatomic reference for computed tomography of the head of the foal. Vet Radiol Ultrasound.

[4] Kl M, Rd P, Tl S, Ts S, Arceneaux B (2000). Computed tomographic imaging of the equine head. Vet Radiol Ultrasound.

[5] Henninger W, Frame E, Willmann M, Simhofer H, Malleczek D, Kneissl S (2003). Ct features of alveolitis and sinusitis in horses. Vet Radiol Ultrasound.

[6] Veraa S, Voorhout G, Klein W (2009). Computed tomography of the upper cheek teeth in horses with infundibular changes and apical infection. Equine Vet J.

[7] Dd C, Er W, Textor J, Mohr F, Scrivani P, Theon A (2012). Computed tomographic appearance of equine sinonasal neoplasia. Vet Radiol Ultrasound.

[8] Veraa S, Dijkman R, Klein W, Van Den Belt A (2009). Computed tomography in the diagnosis of malignant sinonasal tumours in three horses. Equine Vet Educ.

[9] Crijns Cp, Vlaminck L, Verschooten F, Van Bergen T, De Cock He, Huylebroek F, et al.: Multiple Mandibular Ossifying Fibroma In A Yearling Belgian Draught Horse Filly. Equine Vet Educ. 2015;27(1):11–15.

[10] Tietje S, Becker M, Bockenhoff G (1996). Computed tomographic evaluation of head diseases in the horse: 15 cases. Equine Vet J.

[11] Se P (2010). Use of multi-detector computed tomographic angiography in the diagnosis of a parapharyngeal aneurysm in a 6-week-old foal. Equine Vet J.

[12] Hilton H, Puchalski S, Aleman M (2009). The computed tomographic appearance of equine temporohyoid osteoarthropathy. Vet Radiol Ultrasound.

[13] Va L, Sogaro-Robinson C, Reed S (2010). Diagnostic utility of computed tomography imaging in equine intracranial conditions. Equine Vet J.

[14] Pownder S, Scrivani P, Bezuidenhout A, Divers T, Ducharme N (2010). Computed tomography of temporal bone fractures and temporal region anatomy in horses. J Vet Intern Med.

[15] Sogaro-Robinson C, Lacombe V, Reed S, Balkrishnan R (2009). Factors predictive of abnormal results for computed tomography of the head in horses affected by neurologic disorders: 57 cases (2001–2007). J Am Vet Med Assoc.

[16] Jr A, Dd B, Cr B, Md M, Mv C, Rd M (1987). Brain abscess in a horse: diagnosis by computed tomography and successful surgical treatment. Equine Vet J.

[17] Vink-Nooteboom M, Junker K, Van Den Ingh T, Dik K (1998). Computed tomography of cholesterinic granulomas in the choroid plexus of horses. Vet Radiol Ultrasound.

[18] Baum U, Greess H, Lell M, Nomayr A, Lenz M (2000). Imaging of head and neck tumors--methods: Ct, spiral-Ct, multislice-spiral-Ct. Eur J Radiol.

[19] Vanschandevijl K, Gielen I, Nollet H, Vlaminck L, Deprez P, Van Bree H (2008). Computed tomography-guided brain biopsy for in vivo diagnosis of a cholesterinic granuloma in a horse. J Am Vet Med Assoc.

[20] Sm P, Ld G, Wj H, Er W (2007). Intraarterial contrast-enhanced computed tomography of the equine distal extremity. Vet Radiol Ultrasound.

[21] Sm P, Ld G, Cp D, Er W (2009). Use of contrast-enhanced computed tomogrpahy to assess angiogenesis in deep digital flexor tendonopathy in a horse. Vet Radiol Ultrasound.

[22] Sp C, Js M, Re H, Ma M, Al L, O’brien R (2013). Comparison of the diagnostic quality of computed tomography images of normal ocular and orbital structures acquired with and without the use of general anesthesia in the cat. Vet Ophthalmol.

[23] Kishimoto M, Yamada K, Seok J, Shimizu J, Kobayashi Y, Akiba Y (2008). Analysis of blood flow in a third ventricular ependymoma and an olfactory bulb meningioma by using perfusion computed tomography. J Vet Med Sci.

[24] Kromhout K, Gielen I, De Cock H, Van Dyck K, Van Bree H (2012). Magnetic resonance and computed tomography imaging of a carotid body tumor in a dog. Acta Vet Scand.

[25] Ae P, Lenard Z, Mansfield C (2010). Computed tomography diagnosis of eight dogs with brain infarction. Aust Vet J.

[26] Puchalski S (2012). Advances in equine computed tomography and use of contrast media. Vet Clin N Am-Equine.

[27] Ap P, Hc S, Eb H, Js P (2011). Computed tomographic findings in the pituitary gland and brain of horses with pituitary pars intermedia dysfunction. J Vet Intern Med.

[28] Bergman Hj, Puchalski Sm, Saunders J. Intracarotid Contrast-Enhanced Computed Tomography Of The Equine Head. 16th Congress Of The International Veterinary Radiology Association. Bursa, Turkey, 2012.

[29] Ba B, Hindman N, Lee J, Babb J (2007). Multi-detector row ct attenuation measurements: assessment of intra- and interscanner variability with an anthropomorphic body Ct phantom. Radiology.

[30] Ra L, Mr P (1996). Arterial-phase three-dimensional contrast-enhanced mr angiography of the carotid arteries. AJR Am J Roentgenol.

[31] Joshi S, Meyers P, Ornstein E (2008). Intracarotid delivery of drugs: the potential and the pitfalls. Anesthesiology.

[32] Rl E, Morandi F, Daniel W, Paquette J, Daniel G (2007). Comparison of transplenic multidetector Ct portography to multidetector Ct-angiography in normal dogs. Vet Radiol Ultrasound.

[33] Barone R (1996). Anatomie Comparée Des Mammifères Domestiques, Vol. 5 Angiologie.

[34] Nickel R, Schummer A, Seiferle E (2004). Lehrbuch Der Anatomie Der Haustiere, Vol. 3 Kreislaufsystem, Haut Und Hautorgane.

[35] Sisson S, Grossman J (1953). Anatomy of the domestic animals.

[36] Dg M, Pb F, Ke B, Dl H (1999). Anatomic, radiographic and physiologic comparisons of the internal carotid and maxillary artery in the horse. Vet J.

[37] Waldenberger P, Chemelli A, Mallouhi A (2009). Intra-arterial haemodynamic changes during cerebral three-dimensional rotational angiography. Eur Radiol.

[38] Re P, Sm P (2011). Reaction to intraarterial ionic iodinated contrast medium administration in anesthetized horses. Vet Radiol Ultrasound.

[39] De Vries A, Brearley J, Taylor P (2009). Effects of dobutamine on cardiac index and arterial blood pressure in isoflurane-anaesthetized horses under clinical conditions. J Vet Pharmacol Ther.

[40] Gunkel C, Valverde A, Robertson S, Thompson M, Keoughan C, Ferrell E (2004). Treatment for a severe reaction to intravenous administration of diatrizoate in an anesthetized horse. J Am Vet Med Assoc.

[41] Re P, Sm P, Pj P (2008). Hemodynamic and serum biochemical alterations associated with intravenous administration of three types of contrast media in anesthetized cats. Am J Vet Res.

[42] Vance A, Nelson M, Hofmeister E (2012). Adverse reactions following administration of an ionic iodinated contrast media in anesthetized dogs. J Am Anim Hosp Assoc.

